# Flexural Response Comparison of Nylon-Based 3D-Printed Glass Fiber Composites and Epoxy-Based Conventional Glass Fiber Composites in Cementitious and Polymer Concretes

**DOI:** 10.3390/polym17020218

**Published:** 2025-01-16

**Authors:** Abdirahman Ahmed Haibe, Shreya Vemuganti

**Affiliations:** School of Civil Engineering and Environmental Science, University of Oklahoma, 202 W Boyd St., Norman, OK 73019, USA

**Keywords:** 3D printed, fiber-reinforced polymer, glass fiber, flexure, methyl methacrylate, polymer concrete

## Abstract

With 3D printing technology, fiber-reinforced polymer composites can be printed with radical shapes and properties, resulting in varied mechanical performances. Their high strength, light weight, and corrosion resistance are already advantages that make them viable for physical civil infrastructure. It is important to understand these composites’ behavior when used in concrete, as their association can impact debonding failures and overall structural performance. In this study, the flexural behavior of two designs for 3D-printed glass fiber composites is investigated in both Portland cement concrete and polymer concrete and compared to conventional fiber-reinforced polymer composites manufactured using a wet layup method. Thermogravimetric analysis, volume fraction calculations, and tensile tests were performed to characterize the properties of the fiber-reinforced polymer composites. Flexural testing was conducted by a three-point bending setup, and post-failure analysis was performed using microscopic images. Compared to concretes with no FRP reinforcement, the incorporation of 3D-printed glass-fiber-reinforced polymer composites in cementitious concrete showed a 16.8% increase in load-carrying capacity, and incorporation in polymer concrete showed a 90% increase in flexural capacity. In addition, this study also provides key insights into the capabilities of polymer concrete to penetrate layers of at least 90 microns in 3D-printed composites, providing fiber bridging capabilities and better engagement resulting in improved bond strength that is reflected in mechanical performance. The polymer material has a much lower viscosity of 8 cps compared to the 40 cps viscosity of the cement slurry. This lower viscosity results in improved penetration, increasing contact surface area, with the reinforcement consequently improving bond strength. Overall, this work demonstrates that 3D-printed fiber-reinforced polymer composites are suitable for construction and may lead to the development of advanced concrete-based reinforced composites that can be 3D-printed with tailored mechanical properties and performance.

## 1. Introduction

**Figure 1:**
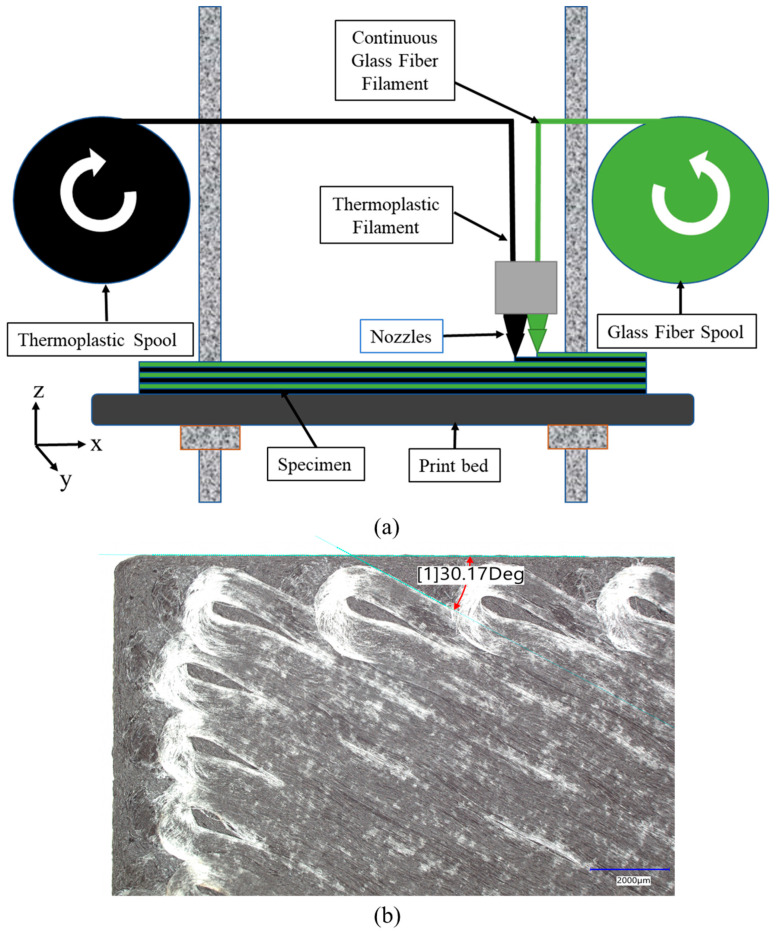
(**a**) Schematic showing 3D printer components. (**b**) Microscopic image taken to show precision of printed fiber angle.

In the field of construction and building, the application of 3D printing technology has already been realized [1,2,3,4,5,6,7,8]. While most of the emphasis is on 3D-printed concrete and cementitious materials, there is also rising momentum for an understanding of the behavior of 3D-printed composites in construction. One of the significant advantages of 3D printing is its ability to create complex, customized geometries. This means that composites can be precisely tailored to fit the specific requirements of a particular structure or component. In addition, various materials can be used for 3D printing, including metals, polymers, ceramics, and composites, catering to factors such as strength, weight, temperature resistance, and cost. Recent advancements in composite 3D printing have led to continuous filament fabrication (CFF) and continuous fiber 3D Printing (CF3D), which overcome the challenges in traditional manufacturing methods for the manufacturing of complex geometries using fiber-reinforced polymer (FRP) composites [9,10,11,12,13,14,15]. FRP is a composite material made of fibers embedded in a polymer matrix. The primary function of fibers in the composite is withstanding the load while providing stability, stiffness, and strength, and the polymer matrix holds the fibers in place, enables the transfer of load, prevents damages from environmental changes, and protects the fibers during manufacturing processes. Additionally, 3D-printed FRP composites with CFF are studied extensively for their tensile properties. Since FRP composites are a typical transversely isotropic material, the orientations of layers with continuous fibers are influential determinants of tensile strength, stiffness, and ductility. Moreover, 3D printing offers great advantages in printing discrete fiber angles over conventional manufacturing techniques by attaining high precision, thus limiting potential deviations and mechanical inconsistencies, as shown in Figure 1.

Using FRP as a composite inside concrete allows for enhanced durability due to its ability to resist corrosion, chemical attack, and environmental degradation, unlike steel composites. It is also shown that the fibers act as micro-composites within the concrete matrix, limiting the width and extent of cracks that may develop under various loading conditions. This improves the serviceability and longevity of the structure. The light-weight characteristics of FRP compared to traditional steel composites simplify handling, transportation, and installation [16,17,18,19,20]. One widely known disadvantage of FRP-reinforced concrete members is that they exhibit poor structural ductility owing to the non-ductile characteristics of the FRP composite and concrete. The structural design of components is based on a ‘fail-safe’ concept, necessitating elements to give a warning prior to failure to prevent catastrophes. In infrastructure applications, the nonlinear behavior of structural components is important to avoid brittle (catastrophic) failure and is required by most design codes worldwide. The ductility of structural elements is required in resilient infrastructure to minimize the loss of capacity, ensure appropriate robustness, and maintain structural integrity after extreme events. If FRP can show ductile behavior, it would make for an attractive alternative in resilient infrastructure. Recently, the potential of 3D-printed FRP to demonstrate pseudo-ductile stress–strain behavior, progressive failure, and delayed strains has been shown, which has furthered interest in 3D-printed-FRP-reinforced concrete (3DP-FRP-RC) components [21,22,23]. With a precise control of fiber orientation, stacking sequence, and the thickness of layers offered by 3D printing technology, it was shown that nonlinear behavior and progressive failure in FRP can be engineered. Control of the surface treatment of fibers was also shown recently for improved interfacial adhesion [24]. Furthermore, recent work has highlighted the residual service life of thermoplastic composites in typical service environments [25]. Such studies facilitate the establishment of durability design guidelines for engineering strengthening applications. Therefore, the use of 3D-printed FRP can have an important role in resilient infrastructure applications, but its behavior inside concrete must be understood first.

This study is the first step toward a pathway of using 3D-printed FRP composites in concrete, which is of very high interest. With advancements in 3D printing, intricate lattice structures, honeycombs, or other complex designs can also be easily fabricated with FRP, providing optimized strength-to-weight ratios and mechanical performance [26,27]. In this study, two types of concrete are considered: cementitious concrete and polymer concrete. Due to the incorporation of polymer resins in polymer concrete as a binder instead of traditional cementitious binders, it is hypothesized that an improvement in flexural capacity can be achieved with FRP composites as opposed to conventional concrete [28,29]. The type of FRP composite included in this study is glass-FRP (GFRP) manufactured using conventional methods and 3D printing technology. To further realize the advantages offered by an engineered internal structure, a 3D-printed GFRP composite with mixed fiber orientations is also used in both the concretes. The flexural responses of 3DP-FRP-RC are evaluated using a four-point bending testing protocol.

## 2. Experimental Materials and Methods

### 2.1. Conventional GFRP Composite

The fabrication process for the conventional GFRP composite followed a vacuum-assisted hand layup technique (VAHT) procedure [4]. One configuration, that is, a unidirectional GFRP composite, was selected with the conventional manufacturing method. A custom weave, unidirectional glass fabric TYFO^®^ SEH-51A oriented in a 0° direction supplied by Fyfe^®^ in a 600 mm wide roll was used, with a minimum weight per sq. yd. of 915 g/m^2^. The composite was prepared using a two-component TYFO^®^ S Saturant Epoxy. The primary component (component A) is a resin system consisting of 4,40-isopropylidenephenol epichlorohydrin. The secondary component (component B) is an aliphatic amine hardener. The supplier-recommended mix ratio is 100 parts of component A to 42 parts of component B by volume and 34.5 parts by weight. Porous release film applied on the top ensured vacuum bag formation with the bottom release film to allow for an efficient vacuum pressure for 24 h. The GFRP composite was heat-cured for 3 days at 60 °C similarly to the manufacturer curing protocol. Post curing, the composite was prepared for tensile testing and volume fraction calculation and included in concrete beams for flexural tests. The properties of the dry fabric and epoxy matrix material are listed in Table 1.

### 2.2. 3D-Printed GFRP Composite

**Figure 2:**
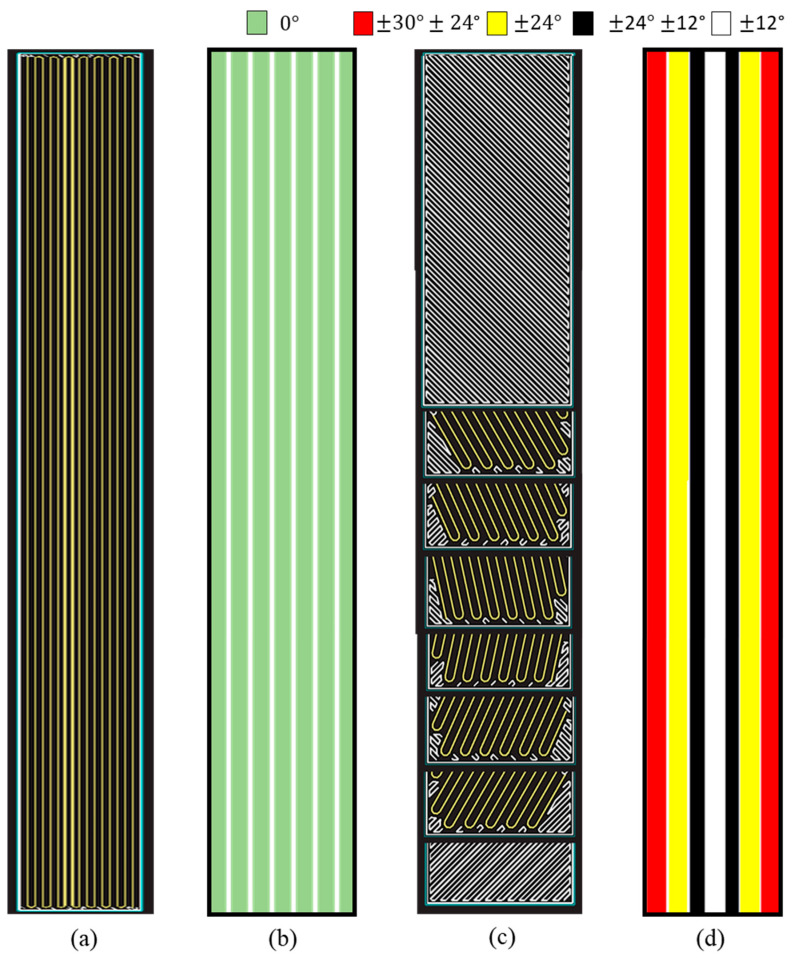
Eiger image and schematic of internal fiber orientations in (**a**,**b**) unidirectional 3D-printed GFRP composite and (**c**,**d**) multidirectional 3D-printed GFRP composite.

The printer used for this study is the Markforged Mark Two™ Billerica, MA, USA enabled with a continuous fiber feature that has the ability to obtain input for fiber orientation through the Eiger^®^ interface. The printer is enabled with a dual printing nozzle head for the thermoplastic nylon-based filament and continuous fiber filament. This allows both the thermoplastic and fiber filaments to be deposited on the same layer during the printing process, as demonstrated in Figure 2a. The specimen design was prepared in AutoCAD and exported as a stereolithography (STL) file into Eiger to prepare for printing. Due to the printer layer resolution of 100 microns, the specimens were printed in 10 layers, resulting in a specimen thickness of 1 mm. The properties of the thermoplastic and continuous fiberglass used in this study are presented in Table 1.

Two configurations of 3D-printed GFRPs were used for this study: a unidirectional 3D-printed GFRP, which consists of continuous fibers printed in a 0° orientation, and a multidirectional 3D-printed GFRP, which consists of continuous fibers printed in different orientations. The multidirectional 3D-printed GFRP design was based on [22,23] to enable a gradual load transfer mechanism between different FRP layers demonstrating characteristic load drops. The design consisted of composites that combined fiber orientations: ±12°, ±24°, ±30° stacked as ±24° ± 30° (2 layers)/±24° (2 layers)/±12° ± 24° (1 layer)/±12° (2 layers)/±12° ± 24° (1 layer)/±24° (2 layers)/±24° ± 30° (2 layers). Refer to the legend in Figure 2 for the layers. The stacking sequence was chosen such that it ensured symmetrically balanced layers in the resulting multidirectional composite with the lowest fiber angle (±12°) in the center, ascending to the highest fiber angle (±30°) at the ends of the composite [21]. A maximum print bed length of 320 mm was available to print specimens, which guided the length of the 3D-printed specimens. The tensile properties of the 3D-printed designs were obtained from past work [21,23,30], as shown in Figure 2.

### 2.3. Concrete Mixing and Fabrication

Two types of mix designs were prepared for this study, including a Portland cementitious concrete (PCC) mix and a Methyl Methacrylate (MMA)-based polymer concrete (PolC) mix. Type I/II Portland cement with a water-to-cement ratio of 0.3 was used for the cementitious concrete mix. The primary components of the polymer concrete design mix were a T-17 polymer concrete liquid component with MMA resin and a T-17 polymer concrete powder component with Benzoyl Peroxide initiator (Transpo Industries, New Rochelle, NY, USA). The nominal maximum size of the fine aggregate used in the cementitious concrete mix and the polymer concrete powder component was 2.36 mm. The well-graded fine aggregate was selected to suit the size of the beams for the flexure test. Specimens were prepared and tested to obtain compressive strength in accordance with ASTM C39-17b [31] for cementitious concrete cylinders and ASTM C579 [32] for polymer concrete cylinders. Specimens were prepared for modulus of rupture (MOR) tests and tested to obtain tensile strength in accordance with ASTM C78 [33] for cementitious concrete and ASTM C580 [34] for polymer concrete. The mix designs for concrete mixes are provided in Table 2.

Specimens were prepared to investigate the flexural response of different composite configurations using a three-point bending test setup. A total of 24 beams were fabricated for the flexural testing program. The beams were 51 mm × 51 mm × 305 mm in dimension. The length of the beam depended on the maximum composite length achievable using 3D printing, which was 320 mm due to print bed limitations. Different composite configurations were investigated for this study with PCC and PolC mixes, with the non-reinforced versions denoted as PCC-NR and PolC-NR, the conventional unidirectional composite versions denoted as PCC-C(U) and PolC-C(U), the 3D-printed unidirectional composite versions denoted as PCC-3D(U) and PolC-3D(U), and the 3D-printed multidirectional composite versions denoted as PCC-3D(M) and PolC-3D(M). For composite placement in the beam molds, side panels with precise rectangular grooves were used. All specimens were demolded after 24 h of casting. PCC specimens were cured in a standard curing room for 14 days. PolC specimens were cured at an ambient room temperature for 7 days.

### 2.4. Flexural Test Setup

**Figure 3:**
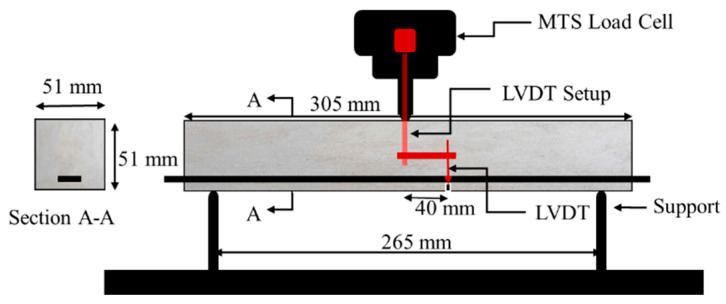
Schematic of three-point bending setup with LVDT placement.

Beams were tested using a three-point bending setup with a span length of 265 mm. The load cell and machine displacement were recorded using a 250 kN MTS 810 hydraulic frame. A displacement rate of 1 mm/min was applied at the midspan location. Deflection was measured using a linear variable displacement transducer (LVDT) at 40 mm from the midspan to avoid damage in case of brittle concrete failure. Midspan deflection was calculated using the recorded deflection at offset using similar triangles. Three beams were tested for each composite configuration with the protocol shown in Figure 3.

### 2.5. Tension Test Setup

Tensile testing on the conventional and 3D-printed GFRP specimens was performed using an MTS 810 hydraulic system with a 250 kN capacity in accordance with ASTM D3039 [35]. An Epsilon extensometer with a 25 mm gauge length was used to record strain in tension test coupons. Five specimens per type were tabbed with CFRP to prevent grip-related damage. The tensile strength was determined with Equation (1), where $\sigma_{c}$ = the stress of the specimen; *A* = the area of the specimen; and *P* = the load applied.

(1) $$\sigma_{c}=\left[\frac{P}{A}\right]$$

### 2.6. Volume Fraction

The volume fraction of conventional and 3D-printed GFRP composites was determined in accordance with ASTM D3171 Procedure G [36]. A compact Split-Tube Furnace with Vacuum Flanges with a maximum temperature of 1200 °C was used to combust the matrix of the GFRP. The fiber volume fraction was determined using Equation (2).

(2) $$\mathrm{Fiber}\mathrm{Volume}\mathrm{Fraction}\;V_{r}=\left[\frac{M_{f}}{M_{i}}\right]\times \left[\frac{\rho_{r}}{\rho_{c}}\right]\times \left[100\right]$$

where $M_{i}$ = the initial mass of the composite specimen before digestion or combustion, g; $M_{f}$ = the final mass of the composite specimen after digestion or combustion, g; $\rho_{r}$ = the density of the composite, g/cm^3^; and $\rho_{c}$ = the density of the specimen, g/cm^3^. The densities of individual constituents were obtained from manufacturer specifications.

### 2.7. Thermogravimetric Analysis (TGA)

For the FRP composite, a 5 mm length of fiberglass continuous filament fabrication spool with a melting temperature of 229 °C was used. To obtain more information about the composition of the fiberglass filament, thermogravimetric analysis was conducted using a TA instruments TGA550 instrument, where 15 mg of fiberglass filament was heated from 30 °C to 700 °C at a heating rate of 20 °C/min in an air environment. The fiberglass content was determined from the residue remaining after the thermal decomposition of the thermoplastic material in the TGA curves. From thermogravimetric analysis, the weight loss measurement of the fiberglass filament was obtained.

### 2.8. Microscopic Analysis

Advanced light microscopy was performed using the Keyence VHX-7000 ultramicroscope at the Samuel Roberts Noble Microscopy Lab (SRMNL) at the University of Oklahoma. The microscope is fully automated and has the ability to capture images with a high magnification of up to 6500×. The precision of fiber orientations obtained using 3D printing technology was studied using microscopic images. In addition, post-testing damage in the flexural test specimens was studied using microscopic analysis and correlated with results to improve the understanding of the flexural behavior of the reinforced concrete beams.

## 3. Results and Discussion

### 3.1. Concrete Properties

Average values of compressive strength and the modulus of rupture for the PCC and PolC mixes were obtained. The average compressive strengths of PCC and PolC were 67.4 ± 1.1 and 59.9 ± 0.9 MPa, respectively. A previous study reported compressive strength values with MMA resin to be 53.9 ± 5.8 MPa [37]. From the four-point bending tests of beams, the modulus of rupture values for the PCC and PolC mixes were determined to be 7.8 ± 0.1 MPa and 23.7 ± 0.72 MPa. Based on the compressive strengths obtained for mix types and curing age, both concrete mixes with similar compressive strengths have been achieved. The tensile strength of the polymer concrete mix measured about three times the tensile strength of Portland cementitious concrete, which is typical [37].

### 3.2. GFRP Composite Properties

**Figure 4:**
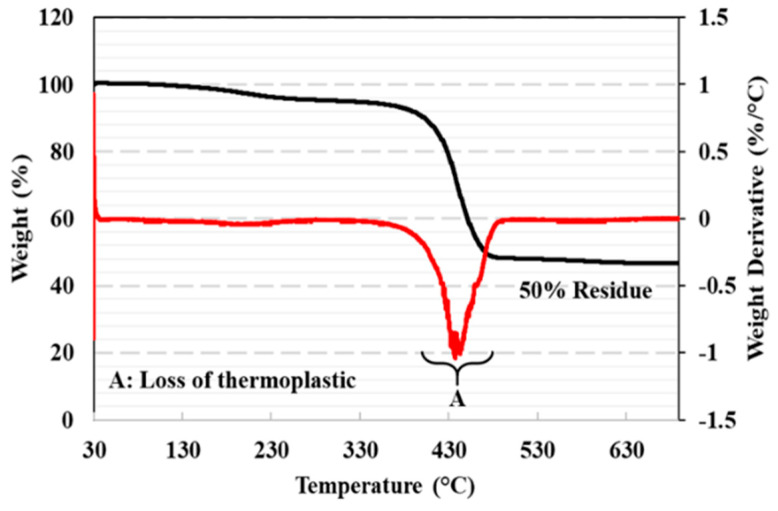
Thermogravimetric analysis of fiberglass filament used for 3D-printed GFRP composite.

Volume fractions of the conventional unidirectional GFRP composite and 3D-printed unidirectional composite were obtained from the tests conducted in this study following ASTM D3171. The average fiber volume fraction of the three specimens of the conventional unidirectional GFRP composite was 42.5% ± 0.4%. For the 3D-printed unidirectional GFRP composite, the average fiber volume fraction was 24.3% ± 0.1%. It can be observed that the fiber volume fraction of the 3D-printed composite is about half that of the composite manufactured using the conventional VAHT process. This is attributed to the presence of thermoplastic in the fiberglass filament used in the 3D printer to allow the melting of the filament onto the print bed. The TGA investigations performed in this study showed the presence of approximately 50% thermoplastic attached to the fiberglass to aid in the melting process and layup of the fiber on the print bed. The decomposition of thermoplastic material in the fiberglass filament occurs predominantly between 380 °C and 475 °C. The initiation of degradation for Nylon 6 has been shown to be at 380 °C, and complete degradation was achieved at 480 °C [38]. The residue (after 475 °C) was in the range of 48% to 55%, which indicates that the fiberglass filaments used in this study for 3D-printed composites had an approximately 50% by weight content of fiberglass, as shown in Figure 4. This agrees well with past work reporting on the weight fraction of fiber present in a fiberglass filament used in 3D printing [23].

**Figure 5:**
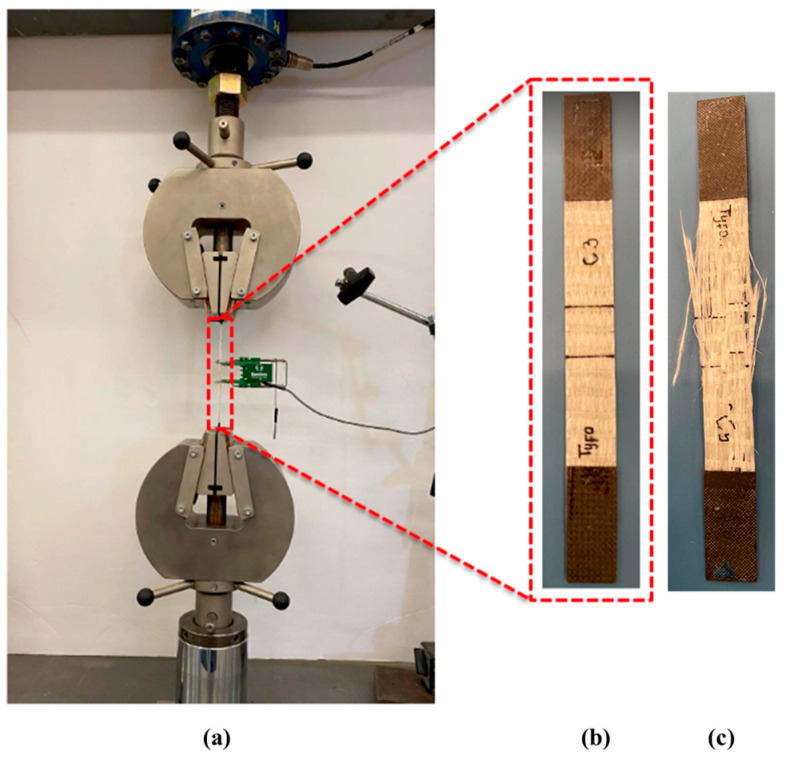
(**a**) Tension test setup; (**b**) conventional unidirectional GFRP composite installed in the setup; (**c**) specimen after failure.

The conventional unidirectional GFRP composite used in this study was tested under tension until the occurrence of broom-like explosive failure that is typical for a 0° fiber direction, as reported in ASTM D3039 [35]. The modulus of the conventional unidirectional GFRP composite is approximately two times the tensile modulus of the 3D-printed unidirectional composite. This is attributed to the higher fiber volume fraction in the former. These results may be reflected in the flexural response of the concrete beams. The 3D-printed multidirectional composite is a low-modulus composite with characteristic load drops unlike the unidirectional composites which experience brittle abrupt failures. A 53% ductility index was expected from the 3D-printed multidirectional composite as opposed to 0% for the other composite types, and the tensile properties of the 3D-printed unidirectional composite and multidirectional composite were obtained from past work [21,22,23]. The properties are listed in Table 3. Figure 5 shows the tension test setup with the brittle failure mode of the tested conventional unidirectional GFRP composite.

### 3.3. Load–Deflection of Concrete Specimens with Reinforcement

**Figure 6:**
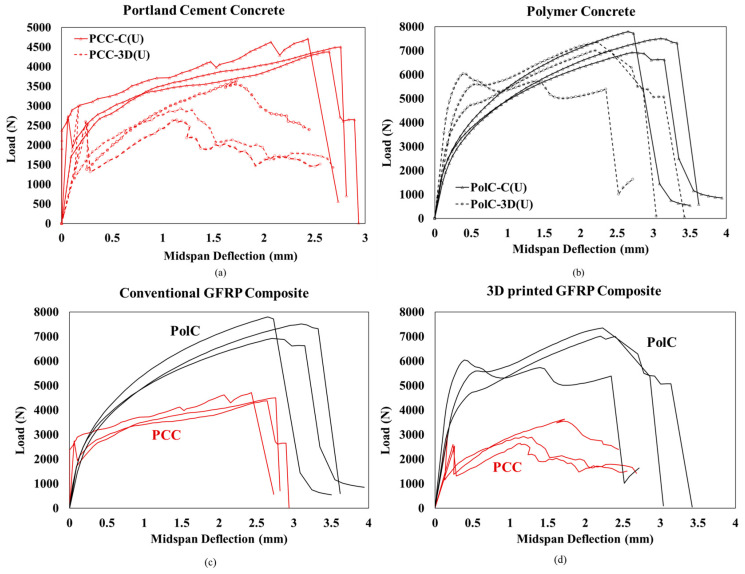
Comparison of the same concrete type for the two different reinforcement types. Flexural response of conventional and 3D-printed GFRP composites in (**a**) Portland cement concrete and (**b**) polymer concrete. Comparison of the same reinforcement type for the two different concretes for three specimens each of Portland cement concrete and polymer concrete with (**c**) conventional GFRP composite and (**d**) 3D-printed GFRP composite.

Looking closely at all three specimens tested for each type of composite in Figure 6, a good agreement can be found, indicating consistency in fabrication, materials, and the testing process. Evidently, the conventional GFRP composite in PCC demonstrates improved stiffness compared to the conventional GFRP composite in PCC. In addition, the yield behavior in PCC is highly evident as opposed to the brittle failure in PolC. The capacity of PolC is higher than that of PCC due to its higher tensile strength. The use of the 3D-printed GFRP composite in PolC improved its stiffness compared to PCC with the 3D-printed GFRP composite. It is important to note that due to the lower stiffness offered by the 3D-printed GFRP composite, PCC has lower stiffness when compared to the conventional GFRP composite. Other important observations include the yielding of PolC with the two different composites. The nonlinear behavior in PolC with the conventional GFRP composite changed to linear behavior followed by yielding when the 3D-printed GFRP composite was used. This may be attributed to the high polymer content in the 3D-printed GFRP composite, as seen in the volume fraction studies. Both nevertheless show brittle failure around the same midspan deflection, indicating that both unidirectional composites have similar behavior.

**Figure 7:**
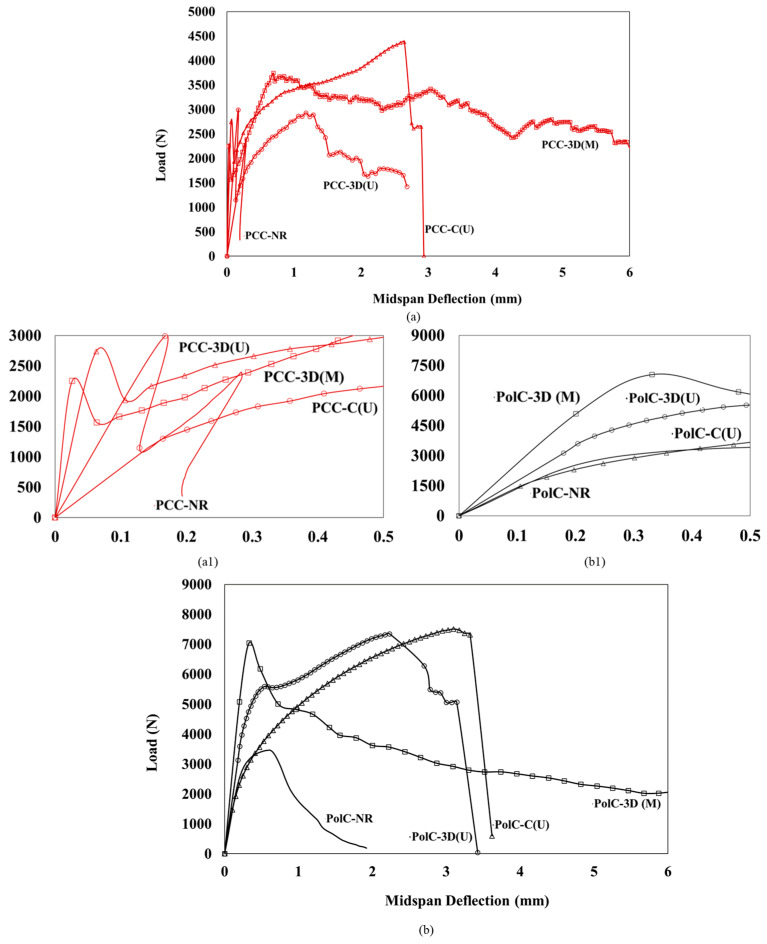
Load–deflection response of (**a**) Portland cement concrete showing up to 0.5 mm deflection in (**a1**) and (**b**) polymer concrete showing up to 6 mm deflection in (**b1**).

Figure 7a shows the load–deflection relationship according to the type of composite used in conventional Portland cement concrete (PCC). Figure 7b shows the load–deflection relationship according to the type of composite used in polymer concrete (PolC). PCC without any composite had a low stiffness and a brittle response to failure. With the incorporation of the conventional unidirectional GFRP, the flexural response demonstrates an increase in capacity and stiffness until brittle failure is observed. A sudden drop in capacity is observed and is gradually picked up due to the composite. With the use of the 3D-printed unidirectional GFRP, a lower stiffness and capacity is observed when compared to the conventional composite. The presence of 3D-printed multidirectional GFRP significantly increased the midspan deflection, and the test was stopped as the boundary conditions of the setup were met. In addition, stiffness was also found to be higher for concrete with the 3D-printed multidirectional GFRP compared to all other types of composites. As shown in Figure 7b, PolC without any composite has a low stiffness and a linear elastic response to a certain extent, after which some non-linearity and brittle failure is seen. With the incorporation of the conventional unidirectional GFRP, the flexural response demonstrated a significant increase in capacity compared to in the case without any composite. The linear elastic response initially followed the same path as the one without any composite. A sudden drop in capacity is observed once peak capacity is achieved. With the use of the 3D-printed unidirectional GFRP, a higher stiffness is observed when compared to the conventional composite. In terms of strength, both composites performed similarly. The presence of the 3D-printed multidirectional GFRP significantly increased the midspan deflection, and the test was stopped as the boundary conditions of the setup were met. In addition, stiffness was also found to be higher compared to in all other types of composites.

**Figure 8:**
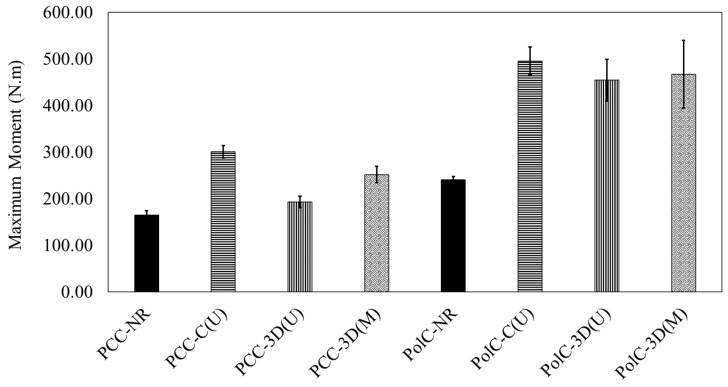
Comparison of load-carrying capacities.

**Figure 9:**
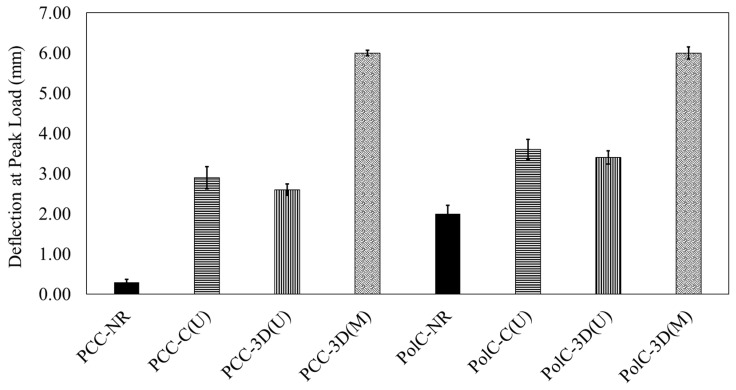
Comparison of maximum deflections measured using LVDT.

In Figure 8 and Figure 9, when comparing PCC with no GFRP composite vs. PCC with the conventional GFRP composite, an 82% increase in flexural capacity is observed. This increase is higher in PolC with no GFRP composite included vs. in PolC with the conventional GFRP composite by about 106%. For the 3D-printed GFRP composite in PCC, a 16.8% increase in flexural capacity is observed as opposed in to PolC, where a 90% increase in flexural capacity solely due to the incorporation of the 3D-printed GFRP composite is observed. To examine statistical significance while comparing the sets of data, Student’s *t*-test (*t*-test) was applied. A 90% confidence interval was used for all the *t*-test. It may be understood from the load–deflection responses that the use of conventional vs. 3D-printed GFRP composites in PCC vs. PolC brought differences to flexural behavior in terms of capacity, response, and maximum midspan deflection.

**Figure 10:**
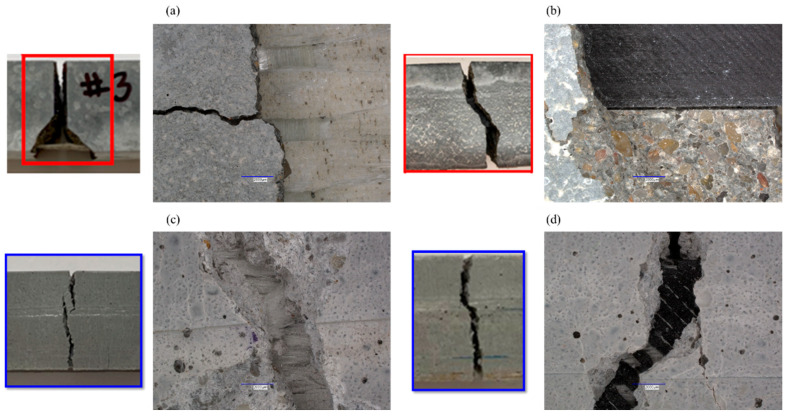
Comparison of crack patterns for (**a**) Portland cement concrete with conventional GFRP composite, (**b**) Portland cement concrete with 3D-printed GFRP composite, (**c**) polymer concrete with conventional GFRP composite, and (**d**) polymer concrete with 3D-printed GFRP composite.

The crack patterns for Portland cement concrete and polymer concrete with the conventional GFRP composite and the 3D-printed GFRP composite are shown in Figure 10. It is remarkable to see the difference between the interactions of the reinforcement types with cementitious concrete vs. polymer concrete. Crack patterns and sizes vary based on the type of concrete and the type of reinforcement. Cone-shaped fractures with horizontal and vertical cracks are observed in PCC with conventional reinforcement. While the cone shape disappears with 3D-printed reinforcement, the cracks still have a combination of inclined and vertical orientations. Vertical cracks grow relatively unrestricted with the negligible toughness of PCC, which is contrary to the behavior in high-tensile-strength polymer concrete. A combination of horizontal and inclined failures is also obtained in polymer concrete with the conventional GFRP composite and the 3D-printed GFRP composite, but the difference is in the nature of the cracks. The opening width of the cracks is much smaller than the observed widths in Portland cement concrete specimens. The growth seems more restrictive, with cracks diverging from the parent cracks. This is attributed to the better engagement of these polymer-based reinforcements with the polymer-based concretes. This evidently confirms the fiber bridging mechanism in the polymer concrete that has led to smaller crack sizes, deformation capacity, ductility, improved load-carrying capacity, and plasticity. Strong fiber bridging is hypothesized to stem from good bond strength between the fabric of the FRP composite and the polymer matrix in concrete, compared with the weak bond between the polymer surrounding the fabric and the cement matrix, as reported by others [23,30,37,39]. Another key observation from the microscopic images that demonstrates the engagement of the polymer concrete with the polymer-based reinforcement is the broom like damage observed in the composites which is not seen in the cementitious concrete. Good impregnation of the FRP improves the bond strength and leads to improved fiber crack bridging and enhanced flexural strength and resistance to crack propagation. The FRP breakage serves as proof of the relatively high bond between the FRP and the polymer matrix in the concrete.

**Figure 11:**
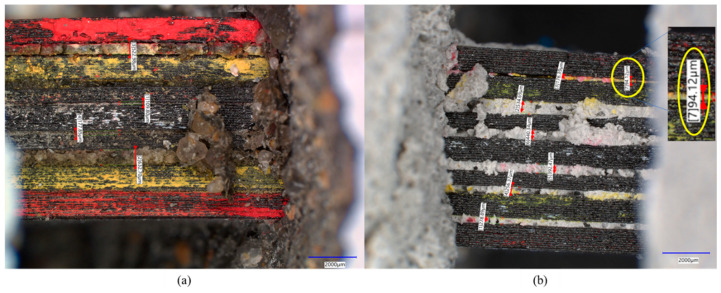
Microscopic image of (**a**) Portland cement concrete and (**b**) polymer concrete demonstrating polymer concrete’s ability to penetrate gaps ranging from 94 micrometers to 440 micrometers.

**Figure 12:**
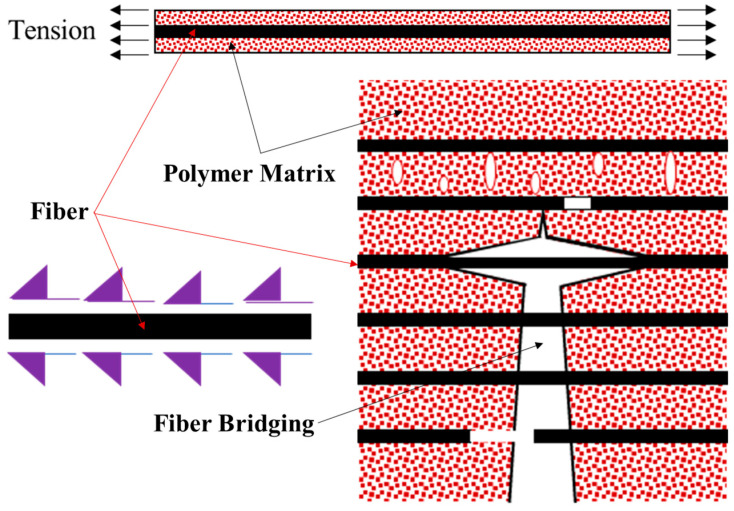
The role of a fiber/matrix interface in the load transfer mechanics and consequently in fracture propagation.

Another remarkable observation from this experimental analysis that demonstrates key proof for the improved bond strength is the microscopic analysis of the specimens of cementitious and polymer concretes with the 3D-printed multidirectional GFRP composite. Proper penetration of the polymer concrete between 3D-printed layers as small as 90 microns is an indication of the high flowability of the fresh polymer concrete slurry shown in Figure 11. This is a key factor for bond strength. The polymer material has a much lower viscosity of 8 cps compared to the 40 cps viscosity of the cement slurry. This lower viscosity results in improved penetration, increasing the contact surface area with the reinforcement and consequently improving bond strength. The connection between penetration, fiber bridging, bond strength, and improvement, and thereby mechanical improvements, is evident through this experimental investigation and analysis. The above-mentioned difference in the penetration and bond between the matrix and the reinforcement results in a difference in the stress transfer in polymer concrete compared to in cementitious concrete. In cementitious concrete, shear forces are typically transferred via friction due to poor adhesion between the FRP and the cementitious matrix shown in Figure 12. On the other hand, polymer concrete seems to transfer the shear forces through friction, relying on the superior adhesion between the FRP and the polymer matrix [37].

The experimental investigation discussed in this paper demonstrates the remarkable capability of polymer concretes to develop improved bond strength and engagement with conventionally fabricated and 3D-printed FRP composites. Many reasons contribute to this engagement and the resulting improved flexural performance, like polymer slurry wettability, viscosity, penetrability, toughness, and the fiber bridging phenomenon. Further research is warranted with other types of fibers and filling fractions of the FRP composite to develop a comprehensive database on the interrelationships between different types of concretes with 3D-printed reinforcements. Production costs and efficiency represent another aspect of future work, since 3D printing is highly efficient for custom, low-volume production with reduced material waste, while traditional methods excel in scalability and speed for large-volume manufacturing. The choice between the two depends largely on the application, production scale, and design complexity.

## 4. Conclusions

This study is an important step toward a pathway of using 3D-printed FRP composites in concrete, which is generating increased momentum in physical civil infrastructure. With advancements in 3D printing, intricate lattice structures, honeycombs, or other complex designs can also be easily fabricated with FRP, providing optimized strength-to-weight ratios and mechanical performance. The TGA investigations performed in this study showed the presence of approximately 50% thermoplastic attached to the fiberglass used in 3D printing to aid in the melting process and layup of the fiber on the print bed. Advancements in state-of-the-art 3D printing technology can improve this number and the resulting effects on mechanical performance. Two types of composites 3D-printed using this filament with unidirectionality and multidirectionality were utilized in cementitious and polymer concretes.

- Overall, the study showed that 3D-printed FRP composites can be a viable reinforcement material in terms of comparable mechanical strength and deflections to failure for cementitious and polymer concretes.
- Compared to concretes with no FRP reinforcement, the incorporation of a 3D-printed GFRP composite in cementitious concrete showed a 16.8% increase in load-carrying capacity, and in polymer concrete, incorporation showed a 90% increase in flexural capacity. This increase is much more evident in polymer concrete when compared to cementitious concrete.
- In addition to demonstrating the viability of 3D-printed FRP composites as flexural reinforcement in concrete, this study also provides key insights into the capability of polymer concrete to penetrate layers of at least 90 microns in 3D-printed composites, providing fiber bridging capabilities and better engagement resulting in improved bond strength that is reflected in mechanical performance.
- The experimental observations in this study create new pathways of 3D-printable reinforcement for different concrete applications. Moreover, 3D printing offers much more flexibility in achieving a combination of fiber orientations, which is highly challenging in conventional FRP fabrication techniques.

## Figures and Tables

**Table 1:** Manufacturer-reported properties of dry fiber and epoxy matrix used for conventional GFRP fabrication; properties of fiberglass filament and thermoplastic used for 3D-printed GFRP composite.

**Conventional GFRP composite**		
	Dry fiber in fabric	Epoxy matrix ^1^
**Properties**	**Manufacturer value**	**Manufacturer value**
Tensile strength **(GPa)**	3.24	0.072
Tensile modulus **(GPa)**	72.4	3.18
Ultimate elongation **(%)**	4.5	5
Density **(g/cc)**	2.55	1.11
**3D-printed GFRP composite**		
	Fiberglass filament	Nylon 66 thermoplastic
**Properties**	**Manufacturer value**	**Manufacturer value**
Density **(g/cc)**	2.55	1.11
Spool volume **(cc)**	150	800
Filament diameter **(mm)**	0.35	1.75
Melting temperature **(°C)**	229	273

^1^ 72 h post curing at 60 °C, tested at 23 °C.

**Table 2:** Mix designs for PCC and PolC mixes. Proportions in kg/m^3^.

Materials	Polymer Concrete	Portland Cement Concrete
Aggregate	2002	2350.6
Water	-	352.7
Superplasticizer	-	9.1
Polymer Resin	251	-
Portland Cement	-	1175.3

**Table 3:** GFRP composite properties.

Properties	Conventional VAHT	3D-Printed Unidirectional ^1^	3D-Printed Multidirectional ^1^
Fiber volume fraction (%)	42.5 ± 0.4	24.3 ± 0.1	-
Tensile modulus (GPa)	29.2 ± 0.8	16.60 ± 0.2	6.4 ± 0.5
Tensile strength (MPa)	526.4 ± 2.6	624 ± 6.5	146 ± 3.3
Strain at first capacity drop (%)	-	-	2.17 ± 0.11
Strain at second capacity drop (%)	-	-	2.6 ± 0.2
Strain at third capacity drop (%)	-	-	3.6 ± 0.2
Strain at failure (%)	1.8 ± 0.13	3.86 ± 0.12	3.68 ± 0.22
Ductility index (%)	0	0	53

^1^ Acquired from [21,22,23].

## Data Availability

The datasets generated during and/or analyzed during the current study are available from the corresponding author on reasonable request.
